# Editorial: Alexithymia: State of the Art and Controversies. Clinical and Neuroscientific Evidence

**DOI:** 10.3389/fpsyg.2019.01209

**Published:** 2019-05-24

**Authors:** Valentina Tesio, Katharina S. Goerlich, Masako Hosoi, Lorys Castelli

**Affiliations:** ^1^Department of Psychology, University of Turin, Turin, Italy; ^2^Department of Biomedical Sciences, Behavioural and Cognitive Neurosciences, University Medical Center Groningen, University of Groningen, Groningen, Netherlands; ^3^Department of Psychosomatic Medicine, Kyushu University Hospital, Fukuoka, Japan

**Keywords:** alexithymia, medical disorders, emotional awareness, negative affectivity, functional disorders

The relevance of alexithymia has grown exponentially in the past decades. It is currently considered a relevant concept for several psychological and physical disorders (Taylor and Bagby, 2004; Di Tella and Castelli, 2013, 2016), and increasing attention is paid to its relationships with other psychological constructs. From a neuroscientific standpoint, an increasing number of studies has been aiming at shedding light on the neuro-anatomical/-functional correlates of alexithymia. The studies included in the present research topic reflect this manifold panorama. Specifically, some studies investigated the relationship between alexithymia and other clinical conditions, i.e., gastrointestinal/hepatological disorders, headache, substance use disorders, autism spectrum disorders (ASD), functional blindness and fibromyalgia. A second block of studies focused on the association between alexithymia and other clinically relevant psychological constructs such as type-D personality, self-harm, emotional recognition and awareness, implicit and explicit positive and negative affectivity. Finally, three studies analyzed neurological/neuro-vegetative aspects.

Among those articles that investigate the prevalence and the impact of alexithymia in medical disorders, Carrozzino and Porcelli systematically reviewed the literature investigating the role of alexithymia in “organic” and “functional” gastrointestinal disorders. Alexithymia, and in particular the difficulty identifying and communicating feelings, were found to be more prevalent in functional gastrointestinal disorders (FGIDs) than in organic ones (i.e., inflammatory bowel disease or liver diseases), with a prevalence of 60% or more vs. 30–50%, respectively. Empirical evidence also suggested that alexithymia is mainly associated to the subjective dimension of health perception, including somatization, symptom persistence, and, above all, negative treatment outcomes. Kano et al. confirmed the association between alexithymia and FGIDs, focusing on the possible mechanism underlying this association. Although empirical evidence proposed several contributing factors, such as somatosensory amplification, visceral hypersensitivity or alteration of the stress response system, the specific mechanism linking alexithymia and FGIDs still remains unclear. The fact that alexithymia is mainly associated to the clinical outcomes related to the subjective dimension of health perception was confirmed by a study on a different functional syndrome, i.e., fibromyalgia. Indeed, Tesio et al. found that alexithymia contributes both directly and indirectly, through the mediation of depressive symptoms, to worsen the impact that this chronic pain pathology has on the patients' quality of life, especially regarding their psychosocial functioning. Another rare functional syndrome, a conversion disorder called non-organic vision loss (NOVL), was investigated by the research paper of Scarpina et al. They found that two patients with NOVL judged themselves as competent in emotional processing on the Toronto Alexithymia Scale (TAS-20; Bagby et al., 1994a,b), but showed a selective deficit in efficiently recognizing the emotion of fear in a behavioral experimental task. These data suggested a mismatch between the patients' subjective evaluation and their actual emotional processing performance.

Natalucci et al. reviewed the few studies on the relation between alexithymia and primary headache (e.g., migraine and tension type headache) in children and adolescents. A general consensus on high levels of alexithymia in this population emerged. The authors suggested that the association between alexithymia and headache could be moderated by insecure attachment or by an incomplete development of emotive competency, which may result in a deficit in emotional regulation and expression. The difficulties in emotional regulation and the lack of emotional awareness, possibly consequent to alexithymia, could compromise the understanding of others' emotions, giving rise to problems in social interaction. The deficit in social competences of people with ASD prompted several authors to investigate the association between alexithymia and ASD. Aiming to evaluate the state of the art, Poquérusse et al. argued that, in spite of the high amount of studies focusing on emotional, social and cognitive features shared by alexithymia and ASD, the nature of this relationship remains unclear. Nevertheless, the scientific evidence seems to support the “alexithymia hypothesis,” i.e., alexithymia, not ASD, would be the main cause of the emotional deficits in ASD. Despite it having been associated with several medical and psychiatric disorders, a recent point of view speculated that alexithymia, rather than characterizing distinct disorders, might be more associated with specific features regarding psychological distress or negative affectivity. Along this line of thought, both the absence of differences between drug-dependent inpatients and psychiatric patients, and the limited ability of alexithymia in predicting treatment outcome led Parolin et al. to question the specificity of alexithymia in substance-dependent patients.

Moving to the articles that focus on the association between alexithymia and other clinically relevant psychological constructs, Suslow and Donges studied its relations with both implicit and explicit positive and negative affectivity. They concluded that there was no substantial relationship between alexithymia and positive affect in healthy adults. However, the difficulties identifying feelings (DIF) factor of the TAS-20 appeared to be associated with an increased conscious experience of negative affect, but not with a heightened disposition for negative affect at an automatic response level. Regarding negative affect, also Epifanio et al. evidenced a strong positive correlation of the negative affectivity factor of the type-D personality with both the DIF and the DDF (difficulty describing feelings) factors of alexithymia. Nevertheless, they confirmed that alexithymia and type-D personality are distinct constructs, with alexithymia being at the base of affect dysregulation underlying the type-D way to cope with emotions. According to Cerutti et al. also non-suicidal self-injury (i.e., the intentional injuring of one's body without apparent suicidal intent) could be regarded as a way to cope with emotions in adolescents with difficulties in identifying and describing feelings. In particular, they proposed a theoretical model in which the difficulty in identifying and describing feelings is a mediator of the associations between non-suicidal self-injury and quality of attachment toward both parents and peers.

Emotional awareness is another construct closely related to alexithymia. Both encompass potential difficulties in identifying and describing one's own and others' feelings, but emotional awareness does not entail limited imaginal ability and externally-oriented thinking. Going further to the partial theoretical overlap, Maroti et al. conducted a meta-analysis of the correlations between the most commonly used measures of alexithymia (TAS-20) and emotional awareness (the Level of Emotional Awareness Scale; LEAS) to explore how the constructs relate to each other empirically. The only small overlap between the two instruments suggested that they measure different aspects of emotional functioning, confirming that alexithymia and emotional awareness are distinct constructs of emotional well-being. The last considered deficit associated with alexithymia is the identification of emotional facial expressions, a crucial mechanism for social interactions. Starita et al. found that participants with high vs. low levels of alexithymia need more emotional intensity to identify static fearful faces, whereas no differences emerged in the identification of dynamic emotional facial expressions. The fact that alexithymia is related to the need for more perceptual information to identify static fearful, but not happy or disgusted facial expressions might be related to partially different brain networks involved.

The latest articles selected for this research topic are focused on the neurobiological correlates of alexithymia. Lischke et al. investigated the association of the vagally mediated heart rate variability (vmHRV) with alexithymia and empathy. vmHRV is a parasympathetical biomarker of the engagement of prefrontal and (para-)limbic brain regions during the regulation of emotional and cognitive processes. Results confirmed that individuals with high vmHRV, which are thus more efficient in recruiting prefrontal and (para-)limbic brain regions implicated in the simulation and interpretation of emotional and mental states, showed more empathy and lower levels of alexithymia than individual with low vmHRV. The introspection-centric simulation theory (IST) predicts that an inability to accurately identify and describe internal affective states may lead to empathy difficulties. Resting on the IST, Valdespino et al. hypothesized that alexithymia may not only be correlated with, but might even cause empathic deficits. Indeed, in their review, they argued that alexithymia and its associated insula pathology could be considered a potential common cognitive and neural marker of empathy deficits across several diagnostic categories. Finally, Goerlich's perspective paper reviewed neuroimaging studies that differentiated the alexithymia facets and dimensions, in order to highlight their dissociable substrates. Results indicated that the DIF and, to a lesser extent, the DDF factors of the TAS-20 usually correlate with emotion-related areas, while the externally oriented thinking factor showed little effect on the function and structure of these brain regions. For example, one large study confirmed a special role of DIF on volumes of the dorsal anterior cingulate cortex, a key region for emotional self-awareness, and other studies linked the DIF factor to a dysfunction of the amygdala during the processing of emotions with negative valence.

Taken together, the articles included in the present research topic clearly highlight the clinical relevance and the complexity of the alexithymia construct, which can be considered transversal to different medical and psychiatric conditions besides being strongly linked to many relevant psychological constructs, like type-D personality. Since psychological factors are more and more recognized as key predictive factors for therapeutic response to treatments (Castelnuovo et al., 2016), further research should investigate the specific role of alexithymia as a potentially relevant outcome predictor, both for medical and psychological conditions.

## Author Contributions

All authors listed have made a substantial, direct and intellectual contribution to the work, and approved it for publication.

### Conflict of Interest Statement

The authors declare that the research was conducted in the absence of any commercial or financial relationships that could be construed as a potential conflict of interest.
